# Hysteresis-Induced Performance Variations and Interfacial Charge Trapping Characteristics in Carbon Nanotube Thin-Film Transistors

**DOI:** 10.3390/nano16140847

**Published:** 2026-07-10

**Authors:** Mingyu Liu, Bo Lai, Hannian Wang, Lele Wu, Wendi Wu, Kai Xu, Yuanchun Zhao

**Affiliations:** State Key Laboratory of Metastable Materials Science and Technology, Yanshan University, Qinhuangdao 066004, China; lmy18633868692@outlook.com (M.L.);

**Keywords:** CNT networks, thin-film transistors, hysteresis, charge trapping, dynamic characteristics

## Abstract

Carbon nanotube (CNT) networks are promising candidate channel materials for thin-film transistors (TFTs). However, the charge trapping characteristics underlying the gate hysteresis effect still remain unclear. Herein, high-performance CNT TFTs with good consistencies were fabricated to investigate the hysteresis-induced performance variations and the dynamic charge trapping/releasing behaviors at different gate biases. Both the subthreshold and suprathreshold characteristics of the TFTs are remarkably changed under different gate sweeping directions. The origin of gate hysteresis was illustrated by comparing the effects of gas desorption and selective re-adsorption, and the adsorbed O_2_ and H_2_O make different contributions related to specific charge trapping characteristics. We further demonstrate that the dynamic charge trapping/releasing processes are governed by the applied gate biases, revealing the equivalency between the positive charge trapping and negative charge releasing processes, and vice versa. The time-dependent degradation of the on-state current has been fitted to perform a statistical analysis based on the measurement results of eight devices. Three characteristic time constants have been determined, corresponding to a multi-step trapping process that may be dominated by dielectric surface trapping and trap-assisted tunneling into the bulk defects in the dielectric layer near the CNTs and those in depth, respectively.

## 1. Introduction

Among various channel materials for advanced thin-film transistor (TFT) technology [1], random semiconducting carbon nanotube (s-CNT) networks are promising and distinctive. Due to their competent carrier mobility, good mechanical properties, and chemical stability [2], CNT networks are suitable for the construction of TFTs with larger channel areas (from several to tens of micrometers), which are competitive with those based on metal oxide semiconductors, low-temperature polysilicon, organics and polymers [3,4]. In particular, they can be processed by solution-based approaches, and thus hold the prospect of low-cost and mass manufacturing of large-area electronics [5]. High-quality CNT networks are characterized by their sub-monolayer nature with a typical surface coverage lower than 20% [6]. Due to an inherently high sensitivity to external environments, CNT TFTs have also been exploited as an attractive platform for chemical and biological sensing [7,8].

Compared with the fully covered TFT channels, the gate-induced electrical field surrounding the isolated nanotubes in the networks is notably higher [9,10]. This promotes significant charge trapping/releasing processes at the nanotube–dielectric interface [11,12], which not only lead to a pronounced gate hysteresis, but also inherently affect the performance of CNT TFT-based sensors. From this point of view, the gate-induced hysteresis should not be reduced or eliminated by vacuum/thermal treatment and subsequent surface passivation [13,14,15,16], but should be well controlled to improve device stability and reproducibility for practical sensing applications. It has been reported that the adsorbed H_2_O and O_2_ molecules play a crucial role in the observed hysteresis of CNT-based devices exposed to the ambient atmosphere [17,18], though their specific effects need to be further clarified by selective adsorption. Meanwhile, the CNT network channel exhibits specific surface charging states at different gate biases [19], which may also have a profound effect on its sensing performance. However, CNT networks (films) deposited for sensing purpose are commonly denser to stabilize the background signals, which in turn suppresses the field effect and, sometimes, leads to a two-terminal device configuration [20,21,22]. Only a few works have investigated the gate effect on the sensing properties of CNT networks [23,24], and the underlying mechanism still remains unclear. High-performance CNT TFTs with good consistency and well-controlled hysteresis could be investigated as a benchmark to present some new insights into the hysteresis evolution and the gate-dependent, dynamic charge trapping characteristics, in which the effect of room-temperature desorption and adsorption of ambient gas molecules is of particular interest due to the common operating conditions of gas sensors.

By controlling the network morphology and the proportion of nanotube bundles, the device performance and consistency of CNT TFTs can be effectively improved [25]. In this work, we demonstrate the hysteresis-induced variations in the key performance parameters averaged over 25 devices, which were properly controlled. The effects of air desorption on the hysteresis behavior was investigated by electrical measurements under continuous vacuum pumping at room temperature, with both the measured on-state current and gate hysteresis exhibiting a decreasing tendency. Then the specific charge trapping characteristics related to selective re-adsorption of O_2_ and H_2_O, respectively, were compared; the re-adsorption of O_2_ leads to a notable recovery of the on-state current with a weak response to hysteresis, whereas that of H_2_O results in a dramatic hysteresis increase. Furthermore, the changes in electrical properties were measured at different gate biases both in the air and in a vacuum, and the corresponding charge trapping/releasing processes were elucidated. Characteristic trapping time constants were also determined by fitting the time-dependent degradation of on-state current based on a triexponential model.

## 2. Materials and Methods

### 2.1. Deposition of s-CNT Networks

A heavily doped Si wafer with a 300 nm thick oxide layer was sliced and used as the substrate. First, the Si/SiO_2_ substrate was cleaned by ultrasonic treatments in acetone, alcohol, and deionized (DI) water, followed by N_2_ drying. The substrate was further treated by oxygen plasma and immersed into a poly-L-lysine solution (0.1% *w*/*v* in water, Sigma-Aldrich, Saint Louis, MO, USA) for 15 min to form an adhesion layer [26]. Then, the substrate was immersed in a high-purity (99.9%) s-CNT aqueous solution (0.01 mg/mL, NanoIntegris Inc., Boisbriand, QC, Canada) to deposit CNT networks. In order to improve the deposition efficiency and reproducibility, the deposition temperature was controlled at 60 °C by a water bath, and the deposition time was 20 min [25]. The obtained samples were sufficiently rinsed with DI water, blown dry using N_2_, and then baked on a hotplate in the air at 240 °C for 30 min to remove the amorphous carbon and the residual chemical agents.

### 2.2. Fabrication and Measurement of CNT TFTs

Source/drain (S/D) electrodes with sandwich structures of Ti/Pd/Au (0.5 nm/20 nm/20 nm) were fabricated by standard photolithography, metal evaporation, and lift-off processes [27], defining a channel length (*L*_CH_) of 20 μm. Additional photolithography and oxygen plasma treatment were performed to remove the CNT networks beyond the channel area and left a channel width (*W*_CH_) of 50 μm. The electrical properties of CNT TFTs were measured both in ambient air and vacuum, by using a Lakeshore TTPX table-top probe station (Lake Shore Cryotronics, Inc., Westerville, OH, USA) combined with a Keithley 4200-SCS semiconductor characterization system. To perform the electrical measurements in vacuum, the chamber of the probe station was continuously pumped for 72 h, which was evacuated to 2 × 10^−6^ Torr after 6 h and then maintained at this pressure. For the selective gas-adsorption measurements, high-purity O_2_ gas and DI water (0.06 mL) were introduced into the sufficiently evacuated vacuum chamber. The O_2_ pressure was maintained at ca. 1 atm, while the concentration of H_2_O was calculated to be that of a relative humidity of 50% at standard atmospheric conditions.

## 3. Results and Discussion

### 3.1. Characterization of the Deposited CNT Networks

**Figure 1:**
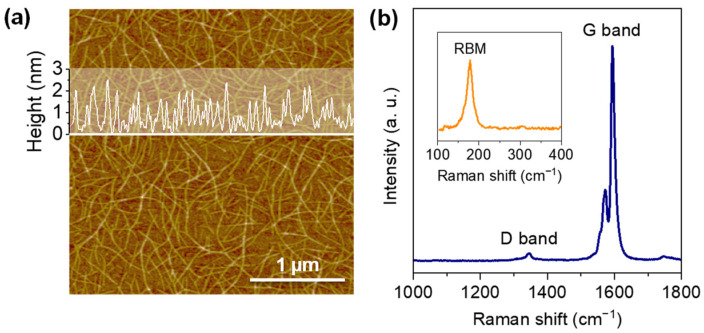
(**a**) AFM image of CNT networks deposited on Si/SiO_2_ substrate. The inset is the corresponding height analysis across the white line. (**b**) Raman spectrum of the deposited CNT networks.

The obtained CNT networks were characterized by atomic force microscopy operated under the tapping mode (AFM, Bruker Dimension Icon, Karlsruhe, Germany) and micro-Raman spectroscopy (Renishaw inVia Raman spectroscope, New Mills, UK) with an excitation source of a 532 nm laser. As shown in Figure 1a, the deposited CNTs are uniform and continuous, with a linear density of ~10.3 counts/μm. The corresponding height analysis reveals that most of the deposited nanotubes are individual with diameters less than 2 nm, while ~22.6% of them are determined to be small bundles with diameters ranging from 2 to 3 nm (inset of Figure 1a). The symmetric G^−^ peak in the Raman spectrum of the CNTs reflects their enriched semiconducting nature (Figure 1b), and the intensity ratio between the G^+^ and D bands (*I*_G_/*I*_D_) is as high as 28.9, revealing their high purity. The radial breathing mode (RBM) peak is centered at 179 cm^−1^, corresponding to an average diameter of ~1.4 nm [28].

It is well known that the purity and morphology of s-CNT networks play the predominant roles in the performance of the fabricated TFTs. Here we used a 99.9% s-CNT aqueous solution to deposit high-quality CNT networks, and the residual surfactants can be readily removed by a sufficient DI water rinsing. The subsequent air annealing process can further oxidize the residual surfactants, amorphous carbon and chemical agents, producing very clean CNT networks (Figure 1a). In addition, the nanotube bundles formed in the networks will deteriorate the device performance; however, this effect has been overlooked in most of the previous studies [29]. By improving the deposition parameters, the proportion of nanotube bundles can be controlled [25], and most of the deposited nanotubes are individual. As a result, the uniform and clean CNT network channels with a controlled proportion of nanotube bundles ensure good device performance and consistency.

### 3.2. Hysteresis-Induced Performance Variations in CNT TFTs

**Figure 2:**
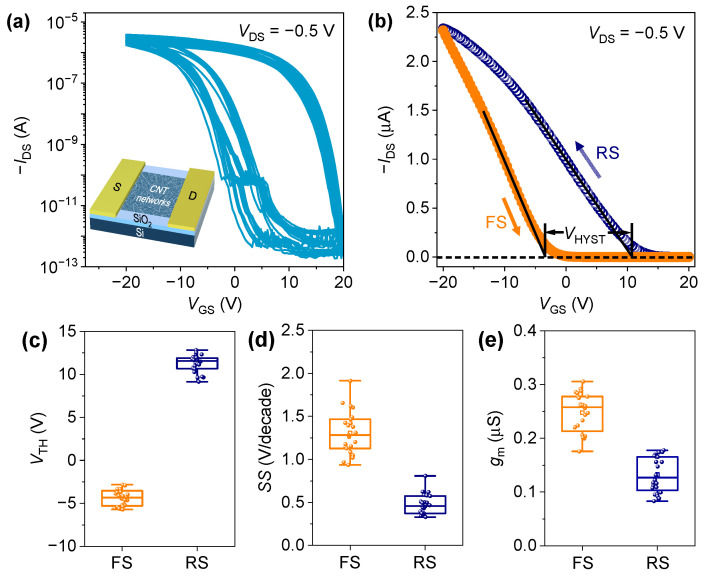
(**a**) Transfer characteristics under a drain voltage (*V*_DS_) biased at −0.5 V for CNT TFTs. For all the devices, *L*_CH_ = 20 µm and *W*_CH_ = 50 µm, respectively. The inset is a schematic of device configuration. (**b**) Transfer characteristics at *V*_DS_ = −0.5 V with measured currents (*I*_DS_) displayed linearly for a CNT TFT, under forward sweeping (FS) and reverse sweeping (RS) of gate voltage (*V*_GS_). Performance variations in (**c**) threshold voltage (*V*_TH_), (**d**) subthreshold swing (*SS*), and (**e**) transconductance (*g*_m_) under different gate sweeping directions are shown across all 25 CNT TFTs.

Figure 2a shows the transfer characteristics of the fabricated TFTs (25 devices from two chips) and the schematic of the device configuration (the inset). The measured on-state current (*I*_ON_) at a gate voltage (*V*_GS_) of −20 V shows a narrow distribution from 1.6 μA to 5.2 μA, with an average on/off ratio of 7.59 × 10^6^. The linear-type transfer characteristics of a representative CNT TFT are shown in Figure 2b, and the gate-induced hysteresis (*V*_HYST_) is defined as the difference between the threshold voltages (*V*_TH_) determined from the two transfer curves under forward gate sweeping (FS, *V*_GS_ from −20 to 20 V) and the reverse (RS, *V*_GS_ from 20 to −20 V) [30], which is found to be 14.47 V for this device. The hysteresis phenomenon also results in a remarkable difference in the output characteristics under different gate sweeping directions (Figure S1).

The performance variations in *V*_TH_, subthreshold swing (*SS*), and transconductance (*g*_m_) derived from the measured CNT TFTs have been summarized. As shown in Figure 2c, the extracted *V*_TH_ values under FS gating ($V_{TH}^{FS}$) are located in the negative *V*_GS_ range, whereas those under RS gating ($V_{TH}^{RS}$) shift into the positive. Both of them are properly distributed due to the good device consistency, corresponding to a controlled hysteresis behavior. Compared to $V_{TH}^{RS}$, the $\left|V_{TH}^{FS}\right|$ values are much smaller (i.e., closer to zero *V*_GS_), and the corresponding weaker gate effect results in relatively poor distribution of *SS*s averaged at 1.3 V/decade (Figure 2d), which is 2.6 times larger than that obtained under RS gating (0.5 V/decade). The calculated *g*_m_ values are plotted in Figure 2e, and the average *g*_m_ under FS gating (0.25 μS) is found to be 1.5 times higher than that under the RS one. These findings highlight the pronounced effect of gate hysteresis on both the subthreshold and suprathreshold characteristics of the CNT TFTs. According to the *g*_m_ values calculated from the FS transfer curves, the nominal field-effect mobility is calculated to be 21.28 ± 2.95 cm^2^/Vs [31], comparable with the reported results of CNT network-based TFTs [32,33,34].

**Figure 3:**
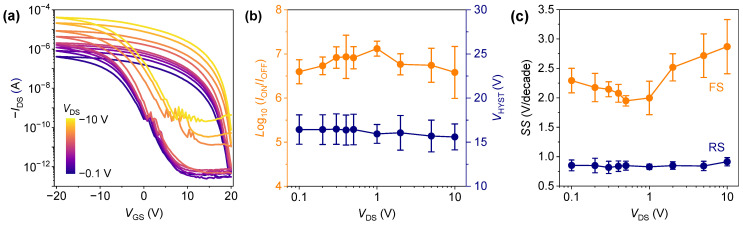
(**a**) Transfer characteristics of a CNT TFT under *V*_DS_ biases from −0.1 V to −10 V. Performance variations of (**b**) on/off ratio and *V*_HYST_, and (**c**) *SS* under different gate sweeping directions averaged across all eight CNT TFTs, respectively.

Figure 3a shows the transfer characteristics of a CNT TFT under various drain-source voltages (*V*_DS_) from −0.1 V to −10 V, revealing its excellent field-effect operating manner [6]. The measured *I*_ON_ exhibits a steady increase from 0.4 μA to 40.0 μA, while the conductance is effectively depleted under a positive *V*_GS_ range from 10 V to 20 V. Interestingly, as the applied *V*_DS_ is less than −1 V, the off-state current (*I*_OFF_) is maintained at pA level. Although relatively poor gate control is observed when further increasing the *V*_DS_, the recorded *I*_OFF_ is still small. The key performance parameters extracted from the transfer curves of eight devices are summarized in Figure 3b,c. The average on/off ratios are found to be higher than 3.81 × 10^6^, with the optimal value of 1.41 × 10^7^ at *V*_DS_ = −1 V. The average *V*_HYST_ shows a slight decrease from 16.43 V at *V*_DS_ = −0.1 V to 15.59 V at *V*_DS_ = −10 V (Figure 3b), because the charge trapping processes can be compensated to a certain extent by the enhanced charge carrier injection under larger *V*_DS_ biases. The derived *SS* values under different gate sweeping directions are consistent with those displayed in Figure 2d, confirming the different gate effects under small *V*_GS_ biases (FS, from −10 V to 10 V) and the sufficiently biased range (RS, from 20 V to 15 V). Moreover, the relationship between the *SS* variations and the applied *V*_DS_ exhibits a direct relevance to that of the average on/off ratios.

### 3.3. Effects of Room-Temperature Gas Desorption and Selective Re-Adsorption

**Figure 4:**
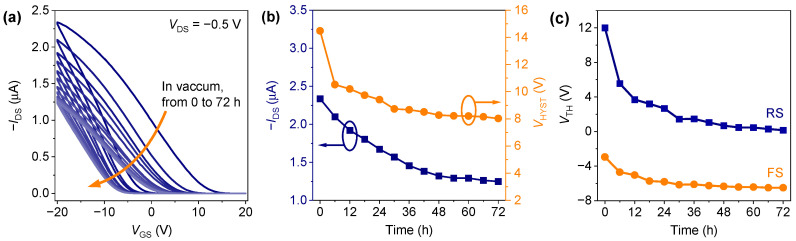
(**a**) Transfer characteristics of a CNT TFT at *V*_DS_ = −0.5 V in a vacuum as a function of pumping time. Time-dependent variations in (**b**) *I*_ON_ at *V*_GS_ = −20 V and *V*_HYST_ extracted from the measured transfer curves, and (**c**) corresponding *V*_TH_ values determined under different gate sweeping directions.

We further measured the transfer characteristics of a CNT TFT in vacuum, where the chamber was evacuated to 2 × 10^−6^ Torr after 6 h. As shown in Figure 4a, the gas desorption process is rather slow, resulting in a continuous negative shift in the *V*_GS_ of the measured transfer curves [35]. The corresponding *I*_ON_ and *V*_HYST_ values were extracted from each measurement and are plotted in Figure 4b. As the chamber is evacuated from 0 to 48 h, the measured *I*_ON_ gradually decreases from 2.34 μA to 1.25 μA, while the *V*_HYST_ drops from 14.47 V to 10.52 V within the initial 6 h and then gradually narrows to 8.03 V. After being pumped for 48 h, both of them become relatively stable. The time-dependent variations in $V_{TH}^{FS}$ and $V_{TH}^{RS}$ are presented in Figure 4c, elucidating that the observed rapid decrease in *V*_HYST_ is indeed dominated by that of $V_{TH}^{RS}$.

It should be noted that, although both of the measured *I*_ON_ and *V*_HYST_ values become relatively stable by maintaining the vacuum for 72 h, the adsorbed gas molecules are hardly completely desorbed, especially for the hydrogen-bonded H_2_O [17]. Therefore, one device chip was annealed at 200 °C for 10 h in a vacuum and then cooled down. As shown in Figure S3, an observed gate hysteresis of ca. 5.63 V is still notable, which is very different from the hysteresis-free conditions observed in the annealed individual nanotube transistor [17], but consistent with those of CNT TFTs [36]. This could be caused by the much larger contact area between the CNT networks and the dielectric surface, as even the adsorbed gas molecules have been nearly completely eliminated, the charge trapping at the nanotube–dielectric interface is still sufficient to induce a gate hysteresis.

**Figure 5:**
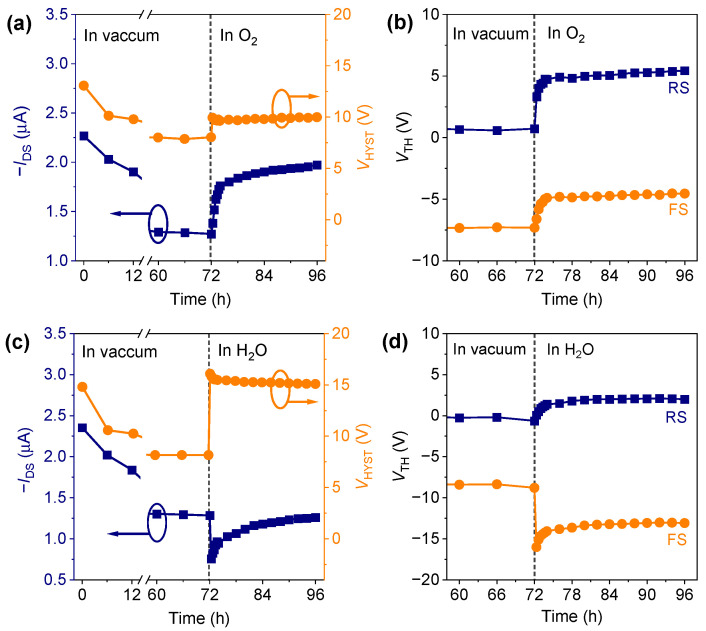
Time-dependent variations in *I*_ON_, *V*_HYST_, and corresponding *V*_TH_ of a CNT TFT measured in a vacuum for 72 h, with (**a**,**b**) high-purity O_2_ gas or (**c**,**d**) 0.06 mL deionized water then introduced into the chamber. The O_2_ pressure was maintained at 1 atm, while the concentration of H_2_O corresponded to that of a relative humidity of 50% under standard atmospheric conditions.

Since the variations in the measured transfer characteristics are dominated by the adsorbed O_2_ and H_2_O molecules [37], we performed a comparative investigation by introducing O_2_ and H_2_O into the sufficiently evacuated chamber in separate experiments. Note that since the CNT networks take the form of a sub-monolayer (Figure 1a), the selective adsorption of gas molecules onto nanotubes should be slow, while the effect of surface adsorption onto the dielectric layer will be more pronounced. As O_2_ was introduced into the chamber after continuous pumping for 72 h, the measured *I*_ON_ at *V*_GS_ = −20 V gradually recovered from 1.27 μA to 1.99 μA over 24 h, while the *V*_HYST_ showed a rapid increase from 8.02 V to 9.73 V and then remained constant (Figure 5a). The continuous O_2_ adsorption onto CNTs induces a p-type doping that positively shifts the transfer curves, and thus the measured *I*_ON_ shows a gradual increase [38]. This effect is also responsible for the positive shifts in both $V_{TH}^{FS}$ and $V_{TH}^{RS}$, as shown in Figure 5b and Figure S2a. Due to the stronger electronegativity of oxygen, however, the O_2_ molecules adsorbed onto the dielectric surface only serve as stubborn negative trap centers that will be effective in the positive *V*_GS_ range, making an additional contribution to the rapid increase in $V_{TH}^{RS}$ at the initial adsorption stage (Figure 5b). This negative charge trapping process leads to the rapid but weak response of *V*_HYST_ to O_2_ adsorption (Figure 5a).

The H_2_O adsorption exhibits different effects on the measured *I*_ON_ and *V*_HYST_. As shown in Figure 5c, after injecting H_2_O into the vacuum chamber the *I*_ON_ directly drops from 1.28 μA to 0.76 μA and then gradually recovers to 1.26 μA, while the *V*_HYST_ shows a dramatic increase from 8.16 V to 16.08 V followed by a slight decrease. The adsorbed H_2_O molecules can serve as both positive and negative trap centers [39]. When *V*_GS_ is sufficiently biased within the negative range (from −10 to −20 V, Figure S2b), the H_2_O molecules adsorbed onto the dielectric surface will facilitate positive charge trapping to screen the gate effect and induce a sharp decrease in both *I*_ON_ and $V_{TH}^{FS}$ (Figure 5c,d); this will be gradually compensated for by the much slower adsorption onto CNTs, leading to these values’ subsequent increases. By contrast, the measured $V_{TH}^{RS}$ in H_2_O addition only exhibits a small increase within the initial 3 h, indicating the limited negative charge trapping process, during which the device is in its off state.

To sum up, O_2_ re-adsorption induces a rapid but small increase in *V*_HYST_ that arises from the variation in $V_{TH}^{RS}$ corresponding to a negative charge trapping process, whereas the dramatic increase in *V*_HYST_ observed in H_2_O is governed by the increase in $V_{TH}^{FS}$ related to positive charge trapping.

### 3.4. Gate-Dependent Characteristics of Charge Trapping and Releasing

During the forward and reverse gate sweepings, both positive and negative charge trapping/releasing processes take place, dependent on specific surface charging states [19]. The positive charge trapping occurs in the large negative *V*_GS_ range, promoting (suppressing) the *I*_DS_ decrease (increase) in the FS (RS) transfer curve, while the positive charge releasing occurs in the relatively small negative *V*_GS_ range, suppressing (promoting) the *I*_DS_ decrease (increase) in the FS (RS) curve. The negative charge trapping/releasing processes exhibit similar behaviors. As a consequence, the gate hysteresis is induced by these dynamic processes.

**Figure 6:**
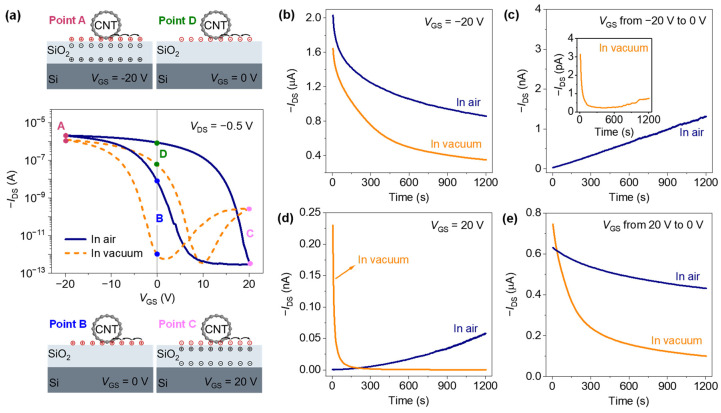
(**a**) Transfer characteristics of a CNT TFT measured at *V*_DS_ = −0.5 V, and schematics of the corresponding surface charging states at the marked *V*_GS_ biases. Time-dependent *I*_DS_ measured in the air and vacuum, respectively, under a specific constant gate bias of (**b**) *V*_GS_ = −20 V then (**c**) switched to 0 V, and (**d**) *V*_GS_ = 20 V then (**e**) switched to 0 V. The *V*_DS_ is biased at −0.5 V for all the measurements.

In order to provide a better understanding of the hysteresis-related charge trapping/releasing processes, the dynamic characteristics of *I*_DS_ were monitored at different *V*_GS_ biases. Figure 6a presents the transfer curves measured in the air and vacuum, in which four specific *V*_GS_ biases have been marked, and the corresponding surface states are schematically illustrated. At a constant *V*_GS_ of −20 V (point A), the induced holes in CNTs are continuously trapped by the adsorbed gas molecules as well as the defective sites in the dielectric layer (a positive charge trapping), and thus the measured *I*_DS_ values show a gradual decrease both in the air and a vacuum (Figure 6b). During the initial 450 s, the decrease in *I*_DS_ in a vacuum is obviously more pronounced than that in the air, whereas the two recorded curves become nearly parallel from 600 to 1200 s, implying a similar slow charging process. Similar results have been reported in previous works measured in the air [35,36,40], whereas that presented in Figure 6b is more characteristic due to the better device performance. The observed time-dependent *I*_DS_ degradation will be discussed later. After the above relaxation, the *V*_GS_ was directly switched to zero (point B, Figure 6a) while the dielectric surface remained in a positively charged state, which triggered the subsequent charge releasing process. The device exhibits p-type conduction in the air, and the residual positive charges are re-injected into the CNTs, leading to an increasing *I*_DS_ (Figure 6c). By contrast, in the vacuum the conductance is completely depleted around *V*_GS_ = 0 V (Figure 6a), where the re-injection of holes consumes the thermally activated electrons in the channel [41]. As a result, the measured *I*_DS_ shows a decrease from 3.14 pA to 0.25 pA in the initial 300 s, and then the thermal conductance is gradually recovered.

Figure 6d depicts the dynamic characteristics of *I*_DS_ at *V*_GS_ = 20 V (point C, Figure 6a), where the device exhibits a completely depleted p-type conductance in the air but a weak n-type conductance in a vacuum. The corresponding negative charge trapping process results in a gradual increase (a rapid decrease) in *I*_DS_ in the air (in vacuum), similar to the trend observed in Figure 6c. When the *V*_GS_ is switched from 20 V to 0 V, the negative charge release will inject electrons into the CNTs, which is responsible for the decreasing tendency of *I*_DS_ (Figure 6d). In particular, the *I*_DS_ measured in a vacuum shows a faster decrease than that in the air, which is similar to the trend measured at *V*_GS_ = −20 V. These findings reflect the equivalency between the positive charge trapping and negative charge releasing processes, and vice versa.

**Figure 7:**
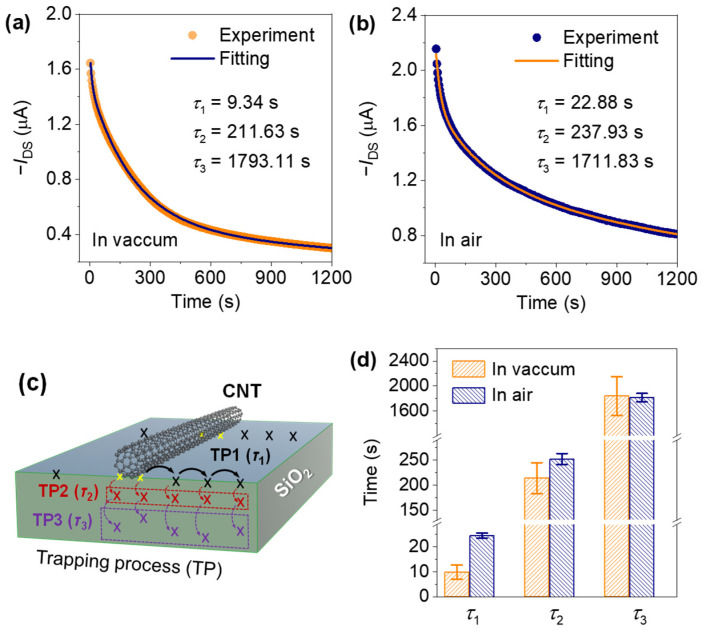
Fitting results of time-dependent *I*_DS_ measured (**a**) in a vacuum and (**b**) in the air, with *V*_GS_ = −20 V and *V*_DS_ = −0.5 V. (**c**) Schematic of charge trapping processes (TPs) corresponding to different decaying components of *I*_DS_. The symbols of “X” represent related charge trapping centers. (**d**) Average characteristic time constants of *τ*_1_, *τ*_2_, and *τ*_3_ extracted from all eight devices.

It is known that the hysteresis in CNT TFTs arises from multi-step charge trapping/releasing processes sequentially occurring at (i) the interface between the CNTs and the dielectric layer as well as the gas molecules adsorbed on the CNTs, (ii) the surface of the dielectric layer surrounding the CNTs, (iii) the bulk defects in the dielectric layer near the CNTs, and (iv) those in depth [36]. The interfacial charging process exhibits a characteristic time less than 1 ms [42], after which the charged traps at the surface are distributed within ~10 nm of the nanotubes at a time scale of 100 ms [35,42], extending to a several-micrometer range after a relaxation over 10 min [43], whereas those at the bulk defects are distributed much slower via trap-assisted tunneling [9]. In this study, the time-dependent variations in *I*_DS_ were measured at a 3.5-s resolution (Figure 6), and thus only processes (ii) to (iv) should be taken into account. Here we choose the characteristic *I*_DS_ degradation measured at *V*_GS_ = −20 V (Figure 6b) for further analyses, which can be well fitted by a triexponential model:

(1) $$I_{DS}=I_{0}-\sum_{i=1,2,3}A_{i}(1-e^{-t/\tau_{i}})$$

where *I*_0_ is the initial drain current at *t* = 0, *τ_i_* (*i* = 1, 2, and 3) are the fitted time constants for different decaying components, and *A_i_* represents the corresponding amplitudes. The fitting results for the decaying *I*_DS_ measured in a vacuum and the air are shown in Figure 7a,b, respectively. Those for other seven devices are plotted in Figure S4, and the fitting results are summarized in Table S1. The experimental data are also fitted by both monoexponential and biexponential models, coupling with a fitting residual analysis (Figure S5), further confirming the rationality of using a triexponential model.

Figure 7c illustrates the proposed charge trapping processes (TPs) corresponding to the above-mentioned processes (ii) to (iv), which may be related to the decaying components of the measured *I*_DS_. The fitted characteristic time constants averaged across all eight devices are plotted in Figure 7d. The average *τ*_1_ is determined to be 9.87 s in a vacuum, 2.07-fold smaller than that determined in the air (20.51 s), which can be attributed to the surface charge trapping (TP1, Figure 7c); the adsorbed gas molecules notably extend this process. Then, both of the surface and interface trapped positive charges are injected into the bulk defects nearby (TP2), corresponding to a longer average *τ*_2_ of 213.07 s in a vacuum, which is slightly shorter than that in the air (252.31 s). The trapped charges then further diffuse toward the deep bulk defects in the dielectric layer (TP3) driven by the large radiating electric field underneath the nanotubes [9,10]; this process should be much slower and unrelated to the surface gas adsorption, and can be described by the similar values of *τ*_3_ averaged at ca. 1800 s.

As for high-speed logic applications [1,2,3], the effect of the above-discussed charge trapping processes (TP1 to TP3, Figure 7c) could be negligible due to their much longer characteristic times. However, they may play important roles in the device’s continuous and reliable gas sensing [7,8]. At a sufficiently biased *V*_GS_, the dielectric surface partially covered by the CNT network channel is indeed fully charged via TP1 [39], which could be modified by competitive adsorption/desorption processes between the target gas molecules and the air and thus affect device performance. If a denser CNT film containing many nanotube bundles is used as the TFT channel, the gate effect will be electrostatically screened, leading to reduced gate hysteresis as well as reduced gas sensitivity, related to the surface charge trapping mechanism [44]. A feasible strategy is to balance the trade-off between sensitivity and reliability by properly controlling the gate hysteresis in CNT TFT-based gas sensors. Moreover, the *I*_DS_ variations induced by TP2 and TP3 are unfavorable for stabilizing the background signals, which need to be suppressed by the rational configuration design of CNT TFTs.

## 4. Conclusions

In summary, we demonstrate the hysteresis-induced performance variations in CNT TFTs with a good consistency, revealing the notable changes in both subthreshold and suprathreshold characteristics under different gate sweeping directions. The adsorbed O_2_ and H_2_O play important roles in the observed gate hysteresis: the former makes a rather weak contribution arising from negative charge trapping, while that of the latter is highly pronounced and related to a positive trapping process. The source-drain current of the device exhibits distinct relaxation features at different gate biases, corresponding to specific charge trapping/releasing processes, and the equivalency between positive charge trapping and negative charge releasing processes—and vice versa—has also been elucidated. The continuous decay of the on-state current has been fitted by a triexponential model, and the derived characteristic time constants provide a reasonable description of the proposed multiple trapping processes. This study highlights the possibility and importance of controlling the gate hysteresis in CNT TFTs and presents a better understanding of the gate-dependent charge trapping/releasing behaviors, offering new opportunities for future device design of TFT-based gas sensors.

## Data Availability

The original contributions presented in this study are included in the article/Supplementary Materials. Further inquiries can be directed to the corresponding author.

## Supplementary Materials

The following supporting information can be downloaded at: https://www.mdpi.com/article/10.3390/nano16140847/s1. Figure S1: Output characteristics of a CNT TFT under (a) forward gate sweeping (*V*_GS_ from −20 to 20 V), and (b) reverse gate sweeping (*V*_GS_ from 20 to −20 V), respectively; Figure S2: Transfer characteristics of a CNT TFT at *V*_DS_ = −0.5 V after continuous vacuum pumping for 72 h, and then by introducing (a) high-purity O_2_ gas and (b) deionized water into the chamber for selective re-adsorption, respectively; Figure S3: (a) Transfer characteristics at *V*_DS_ = −0.5 V of a CNT TFT measured in the air, in vacuum, and after annealing, respectively. (b) Corresponding *V*_HYST_ averaged over 5 devices; Figure S4: Fitting results of time-dependent *I*_DS_ of the other seven devices measured in air and vacuum, with *V*_GS_ = −20 V and *V*_DS_ = −0.5 V; Table S1: Characteristic time constants determined form the fitting results of time-dependent *I*_DS_ degradation across all 8 devices; Figure S5: Fitting results based on monoexponential and biexponential models, and the corresponding residual analyses of the same time-dependent *I*_DS_ data shown in Figure 6b measured (a,b) in vacuum and (c,d) in the air, with *V*_GS_ = −20 V and *V*_DS_ = −0.5 V.
