# Supplementary material for: Hysteresis-Induced Performance Variations and Interfacial Charge Trapping Characteristics in Carbon Nanotube Thin-Film Transistors
Source: Nanomaterials (Basel). 2026 Jul 10;16(14):847. doi: 10.3390/nano16140847 (PMC13414926; doi:10.3390/nano16140847)
Supplement: Supplementary file 1 [file nanomaterials-16-00847-s001.zip › nanomaterials-4375486-supplementary.pdf]

Supplementary Materials for

Hysteresis-Induced Performance Variations and Interfacial Charge Trapping Characteristics in Carbon Nanotube Thin-Film Transistors

Mingyu Liu, Bo Lai, Hannian Wang, Lele Wu, Wendi Wu, Kai Xu and Yuanchun Zhao*

State Key Laboratory of Metastable Materials Science and Technology, Yanshan University, Qinhuangdao 066004, China

* Correspondence: yzhao@ysu.edu.cn

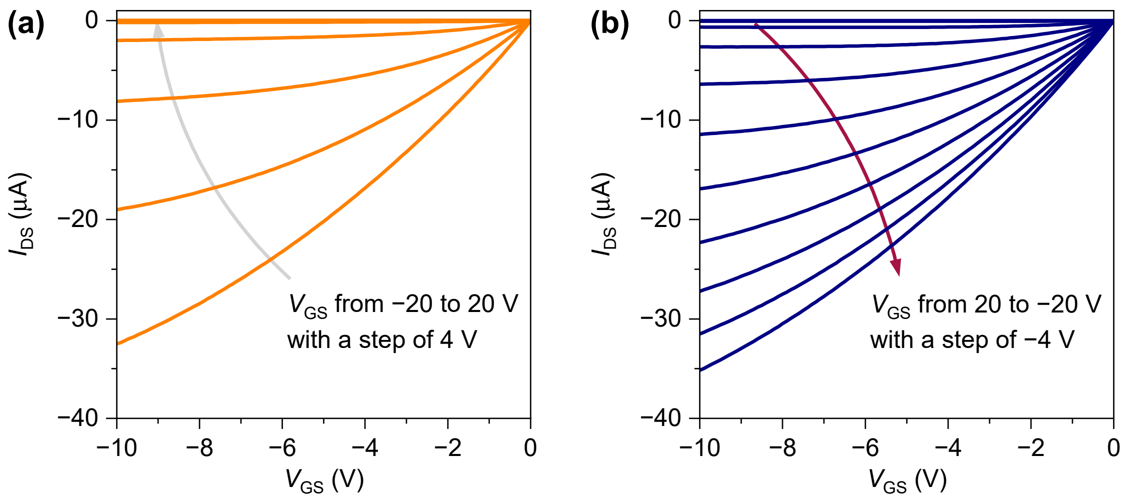

Figure S1. Output characteristics of a CNT TFT under (**a**) forward gate sweeping (V_{GS} from −20 to 20 V), and (**b**) reverse gate sweeping (V_{GS} from 20 to −20 V), respectively.

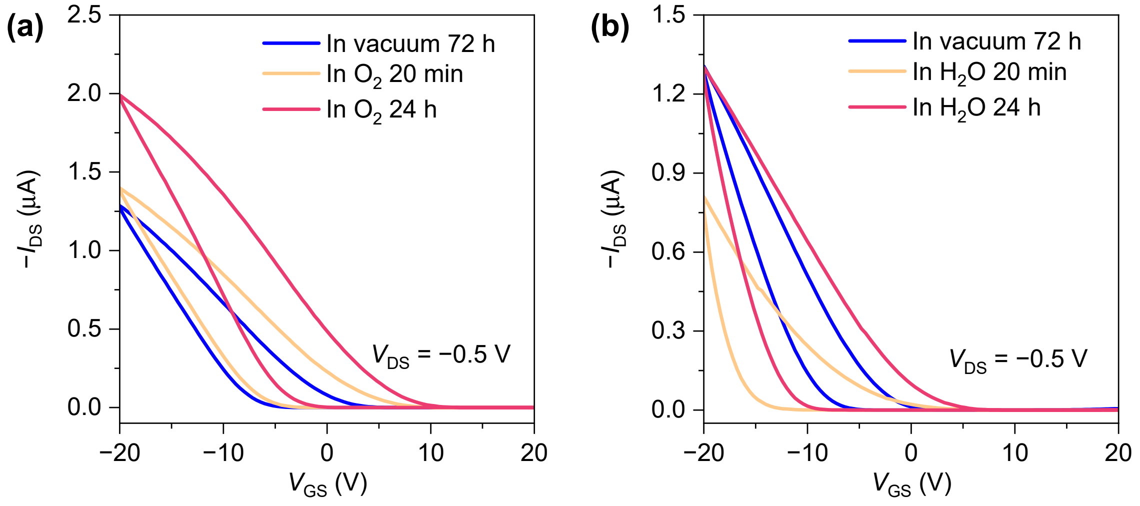

Figure S2. Transfer characteristics of a CNT TFT at V_{DS} = −0.5 V after continuous vacuum pumping for 72 h, and then by introducing (**a**) high-purity O_2 gas and (**b**) deionized water into the chamber for selective re-adsorption, respectively.

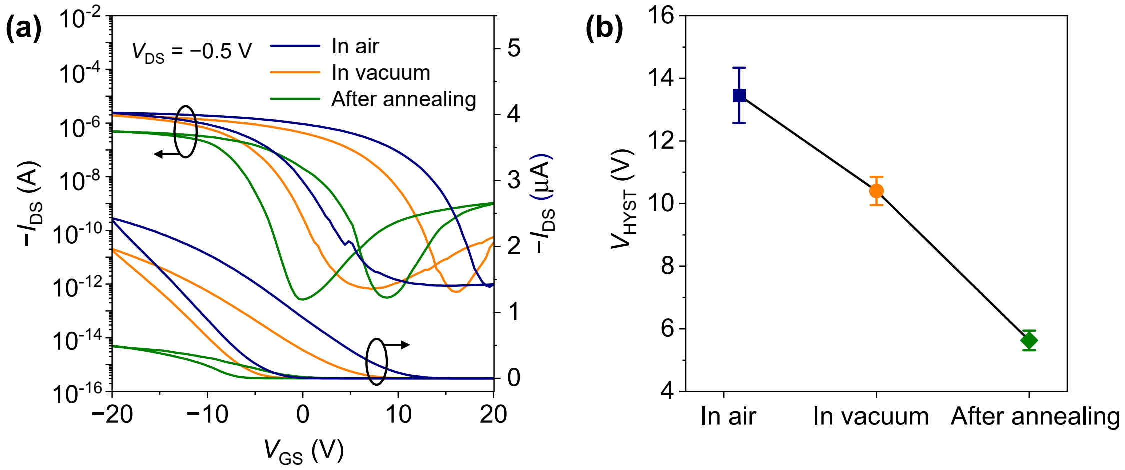

Figure S3. (**a**) Transfer characteristics at V_{DS} = −0.5 V of a CNT TFT measured in the air, in vacuum, and after annealing, respectively. (**b**) Corresponding V_{HYST} averaged over 5 devices.

Note: In order to perform nearly complete gas desorption, the device chip was kept in the chamber and evacuated for 10 h, and the measured transfer curve is plotted in Figure S3 as "In vacuum". Then, it was *in-situ* annealed on the sample plate at 200 °C for 10 h and cooled down to room temperature. The transfer characteristics were still measured in vacuum, and the results are plotted Figure S3 as "After annealing".

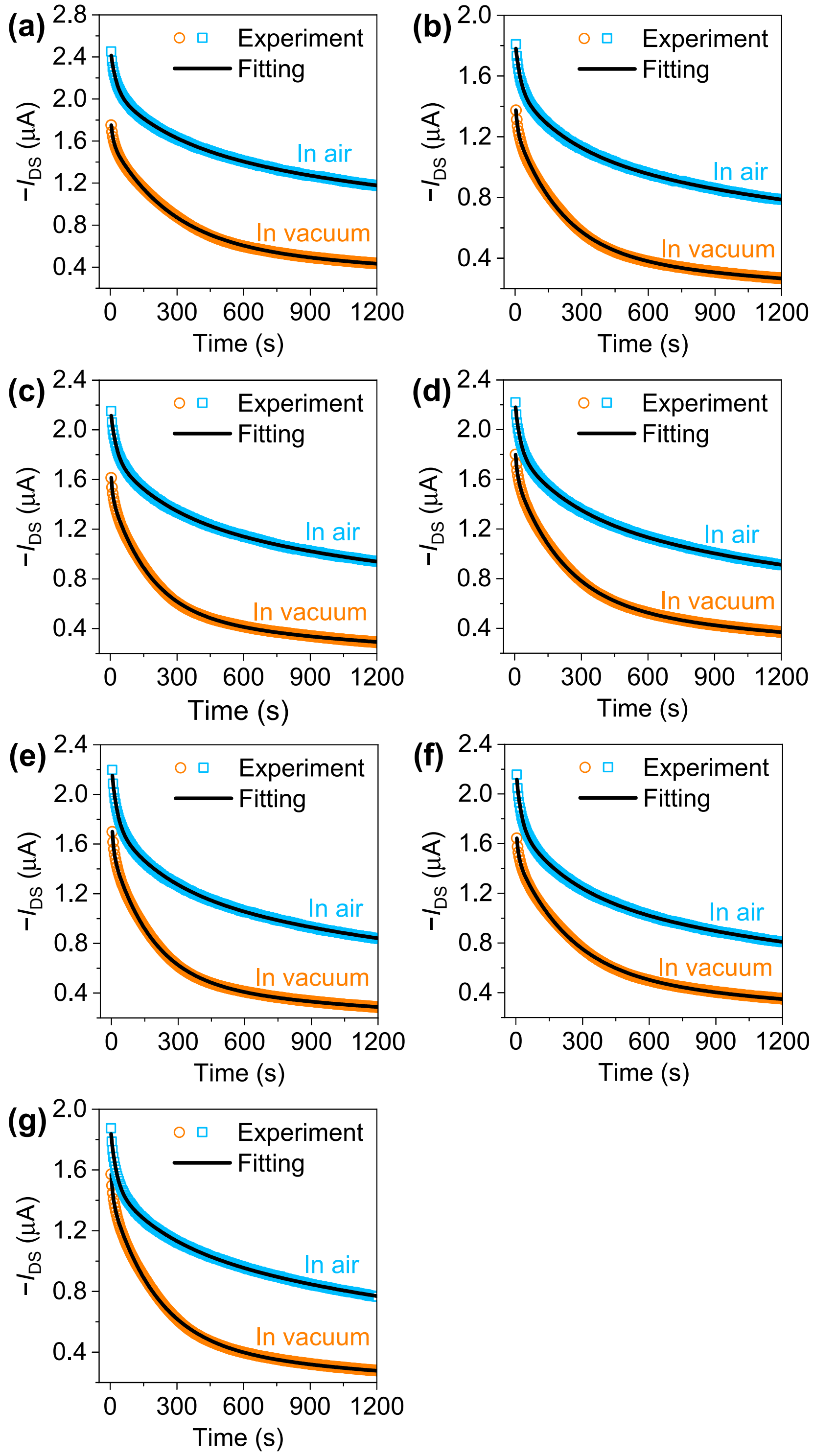

Figure S4. Fitting results of time-dependent I_{DS} of the other seven devices measured in air and vacuum, with V_{GS} = −20 V and V_{DS} = −0.5 V.

Table S1. Characteristic time constants determined form the fitting results of time-dependent I_{DS} degradation across all 8 devices.

Device	In vacuum			In air		
	τ_1 (s)	τ_2 (s)	τ_3 (s)	τ_1 (s)	τ_2 (s)	τ_3 (s)
1	10.40	270.04	2439.86	25.58	268.02	1823.04
2	8.25	210.24	1894.36	23.26	244.56	1803.15
3	7.48	175.56	1488.22	24.75	260.05	1852.58
4	10.04	215.67	1675.45	24.12	257.86	1882.58
5	8.23	181.27	1548.36	24.82	238.87	1708.77
6	9.66	241.22	2072.82	24.71	259.81	1851.60
7	8.83	205.59	1776.31	25.98	251.38	1863.69

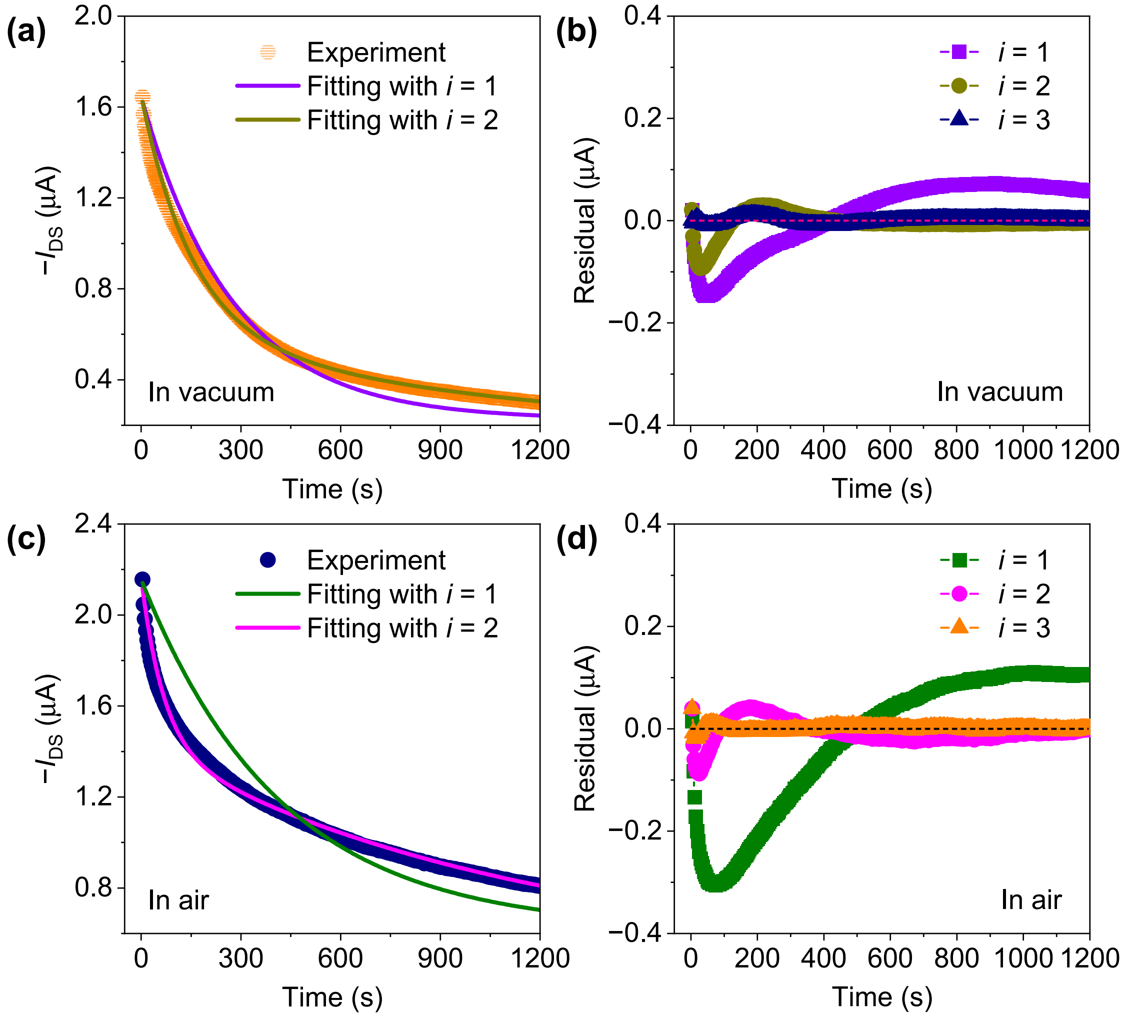

Figure S5. Fitting results based on monoexponential and biexponential models, and the corresponding residual analyses of the same time-dependent I_{DS} data shown in Figure 6b measured (**a**,**b**) in vacuum and (**c**,**d**) in the air, with $V_{GS} = -20$ V and $V_{DS} = -0.5$ V.
